# A Case of a Large Seminal Vesicle Cyst Associated with Ipsilateral Renal Agenesis

**DOI:** 10.1100/tsw.2008.65

**Published:** 2008-04-20

**Authors:** Apostolos P. Labanaris, Vahudin Zugor, Bernd Meyer, Reinhold Nützel, Reinhard Kühn

**Affiliations:** ^1^ Department of Urology, Martha Maria Medical Center, Nurnberg, Germany; ^2^ Department of Urology, University of Erlangen Medical Center, Erlangen, Germany

**Keywords:** seminal vesicle cyst, renal agenesis, incidence, treatment

## Abstract

The diagnosis of seminal vesicle cysts is often delayed or missed as a result of both their rarity and wide spectrum of potentially confusing clinical and imaging findings they can produce. Although rare, they should be considered in men, especially with a history of renal agenesis, who exhibit o inexplicable irritable voiding symptoms, perineal discomfort or other genitourinary complaint of unclear etiology. We introduce such a case, and discuss its symptoms, radiological findings and its therapeutic approach.

## INTRODUCTION

Congenital anomalies of the seminal vesicles can be categorized into abnormalities of number, structure, maturation, and position. In practice, anomalies of the structure are most commonly encountered in the form of cysts[1]. Seminal vesicle cysts (SVC) are seen in the population in a ratio of less than 0.005% and are usually associated with ipsilateral renal agenesis[2]. The diagnosis of SVC is often delayed or missed as a result of both their rarity and wide spectrum of potentially confusing clinical and imaging findings they can produce. We introduce a case of SVC associated with ipsilateral renal agenesis and discuss its symptoms and radiological findings, as well as its therapeutic approach.

## CASE REPORT

A 27-year-old man presented to our department with vesical irritability accompanied by repeated bladder infections the previous 2 years, as well as postcoital discomfort. He had been previously treated with multiple courses of antibiotics without symptom resolution as he was thought to have prostatitis. His anamnesis revealed a right renal agenesis as well as treated ipsilateral cryptorchidismus. The physical examination of the abdomen did not exhibit any pathological signs. The rectal examination revealed an elastic, soft, tender prostate of 20 ml in size. Prostatitis was suspected. Expressed prostatic secretions and urinary sediment voided after prostatic massage did not reveal any white blood cells. Luteinizing hormone, follicle-stimulating hormone, prolactin, and testosterone were within the normal range.

Sonography exhibited a solid hypoechoic, well-defined, and homogeneous mass projecting in the bladder (Fig. 1). Dynamic contrast-enhanced endorectal magnetic resonance imaging (DCE-eMRI) demonstrated a large cyst that appeared to be arising from the right seminal vesicle and displacing the bladder anterior and medial. Its signal intensity reflected to that of fluid (Figs. 2 and 3). The preoperative diagnosis made then was that of SVC associated with ipsilateral renal agenesis. In order to exclude some other pathology of the bladder, urethrocystoscopy was performed and showed an edematous seminal colliculus and elevation near the bladder neck; the right vesical hemitrigone was missing. With the manipulations of

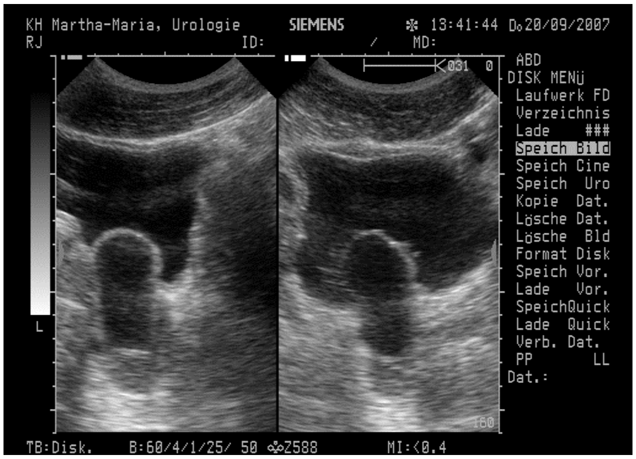

**FIGURE 1.** A solid hypoechoic, well-defined, and homogeneous mass projecting in the bladder.

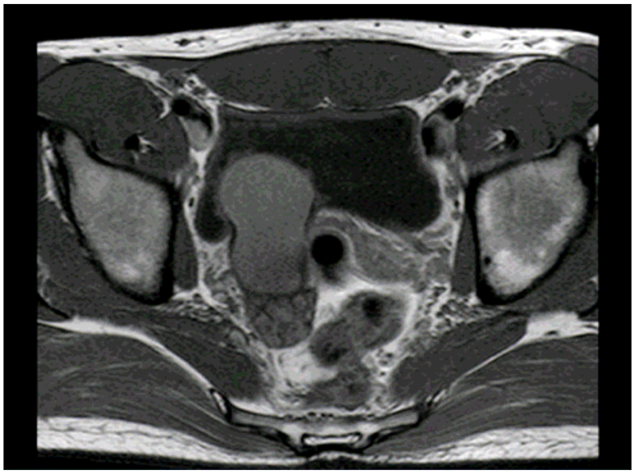

**FIGURE 2.** DCE-eMRI exhibiting a SVC.

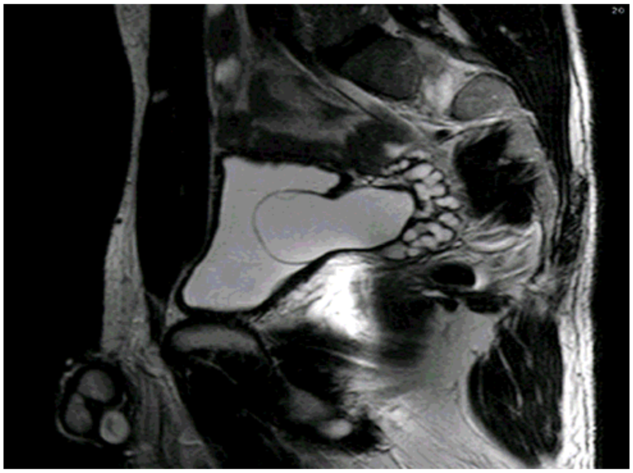

**FIGURE 3**. DCE-eMRI exhibiting a SVC.

the cystoscope, the SVC was ruptured so a transurethral resection of the remaining intravesical SVC was performed. The pathologic examination demonstrated a fibrous-walled cyst with focal dystrophic calcification, nonspecific inflammation, and focal benign seminal vesicle tissue. The aspirate demonstrated spermatozoa, thus wrapping up the diagnosis of SVC. After 120 days, the patient is free of symptoms and no signs of retrograde ejaculation are evident.

## DISCUSSION

Congenital SVC can be subcategorized as isolated cysts, cysts associated with upper urinary tract anomalies, and cysts associated with autosomal-dominant polycystic kidney disease. SVC are associated with ipsilateral renal agenesis or dysplasia in two-thirds of patients. The male reproductive and urinary systems are closely related embryologically and anatomically, explaining the coexistence of renal and reproductive duct anomalies. Their significance lies in their frequent association with abnormal development of other mesonephric derivatives. Presumably, the maldevelopment of the distal mesonephric duct and faulty ureteral budding leads to renal agenesis or dysplasia, and atresia of the ejaculatory duct leads to obstruction and cystic dilatation of the seminal vesicle[3]. Ipsilateral absence or cryptorchidismus of the testis, as well as hemivertebra, can also be present[4]. Zinner[5] first described the association of SVC with ipsilateral renal agenesis in 1914, and noted the association of these cysts with other genital anomalies.

When smaller than 5 cm in diameter, they are usually discovered incidentally since they are asymptomatic[6]. Symptomatic congenital cysts typically present between the ages of 18 and 30 years, the period of maximum sexual and reproductive activity, when seminal fluid accumulates in the seminal vesicles as a result of stenosis of the ejaculatory duct[7]. Symptoms mostly develop due to the irritation of adjacent organs by the enlarged and inflamed cyst. Bladder irritation causes urgency, frequency, dysuria, and hematuria. Cyst distention may cause perineal, suprapubic, flank, pelvic, and scrotal pain; hematospermia; postcoital pain or discomfort; as well as painful defecation. Obstruction of other mesonephric duct derivatives may lead to epididymitis or prostatitis. Many patients, as well as the mentioned patient, have been treated with multiple courses of antibiotics without symptom resolution. Occasionally, infertility may be the chief complaint.

Although congenital anomalies of the seminal vesicles are rare, they are likely to be increasingly encountered because of the continued growth in the use of ultrasound, CT, and MRI. In ultrasonography, SVC typically appear as paramedian cystic structures. Such cysts should be distinguished from Müllerian duct cysts, which are characterized by a midline location with normal seminal vesicles on either side[8]. In a sonographic screening study of the kidneys in 280,000 children in Taipei, the frequency of presumed SVC with ipsilateral renal agenesis or dysplasia was 0.0046%[2]. On CT, they appear as a well-defined retrovesicular mass of water or near-water attenuation that arises from the seminal vesicle cephalic to the prostate gland[3]. On MRI, SVC are of variable signal intensity on T1-weighted images, are generally of fluid signal intensity on T2-weighted images, and are nonenhancing after intravenous gadolinium administration. Increased T1-weighted intensity is thought to reflect hemorrhage or an increased concentration of proteinaceous fluid[3]. MRI is a better tool for accurately defining anatomic relationships when one is planning to excise the SVC or if one is considering a difficult differential diagnosis because of its multiplanar imaging capability, superior soft-tissue contrast, and lack of ionizing radiation. MRI may also help to depict the opening of the associated ectopic ureter[9].

In this patient, DCE-eMRI was performed and made the diagnosis, as it is well known that endorectal functional MRI is the best imaging modality of the prostate. Cystoscopy may identify an absent ipsilateral hemitrigone, intravesical cyst protrusion, and any other anatomical abnormality of the bladder[10]. SVC contain viscous pale white fluid and are lined with a cuboidal or flattened epithelium with a fibrous wall[11]. The presence of spermatozoa in the aspirate differentiates SVC from other abnormalities.

Differential diagnosis must be made from cystadenoma and papillary adenoma, both benign tumors of the seminal vesicle, which may mimic simple cystic enlargement in both presentation and imaging[12]. Other possible differential diagnoses include Müllerian duct cysts, diverticula of the ejaculatory ducts or ampulla of the vas, prostatic cysts, and malignant tumors of the vesicles, both primary (adenocarcinoma and sarcoma) and secondary, caused by spread from the bladder, prostate, rectum, and lymphomas.

Incidentally discovered SVC that do not cause pain or functional impairment should be followed without intervention. When the patient is symptomatic, surgical excision is the treatment of choice. Treatment includes transurethral unroofing, transurethral resection of the ejaculatory canal, transurethral endoscopic aspiration, transabdominal aspiration, transperineal aspiration, transrectal aspiration, and open surgery. Abdominal, retroperitoneal, perineal, or laparoscopic approaches can be chosen as surgical options. Transrectal aspiration may transiently relieve symptoms and allow the instillation of contrast material to establish the diagnosis. Simple cyst drainage is associated with the risk of recurrence, return of symptoms, and possible infection[12]. Cystoscopy and transurethral resection of the ipsilateral ejaculatory duct or intravesical cyst unroofing are minimally invasive approaches that have met with satisfying success in the literature, but can result in retrograde ejaculation. Laparoscopy has been advocated as the best minimally invasive technique for the surgical treatment of seminal vesicle pathology[10].

The conclusion of this case report is that SVC, although rare, should be considered in men, especially with a history of renal agenesis, who exhibit inexplicable irritable voiding symptoms, perineal discomfort, or other genitourinary complaint of unclear etiology. Evaluation should include digital rectal examination, transrectal and transabdominal ultrasound, MRI, and cystoscopy. Although the therapy of choice is laparoscopy, transurethral resection can also be performed with a reduced risk of complications.
